# Is there a causal link between intracellular Na elevation and metabolic remodelling in cardiac hypertrophy?

**DOI:** 10.1042/BST20170508

**Published:** 2018-07-03

**Authors:** Dunja Aksentijevic, Brett A. O'Brien, Thomas R. Eykyn, Michael J. Shattock

**Affiliations:** 1 School of Biological and Chemical Sciences, Queen Mary University of London, G.E. Fogg Building, London, U.K.; 2 King's College London, School of Cardiovascular and Medical Sciences, British Heart Foundation Centre of Research Excellence, St Thomas Hospital, London, U.K.; 3 Department of Imaging Chemistry and Biology, School of Biomedical Engineering and Imaging Sciences, King's College London, St Thomas’ Hospital, London, U.K.

**Keywords:** bioenergetics, cardiac hypertrophy, metabolism, mitochondrial dysfunction, sodium

## Abstract

Alterations in excitation–contraction coupling and elevated intracellular sodium (Na_i_) are hallmarks of pathological cardiac remodelling that underline contractile dysfunction. In addition, changes in cardiac metabolism are observed in cardiac hypertrophy and heart failure (HF) that lead to a mismatch in ATP supply and demand, contributing to poor prognosis. A link between Na_i_ and altered metabolism has been proposed but is not well understood. Many mitochondrial enzymes are stimulated by mitochondrial calcium (Ca_mito_) during contraction, thereby sustaining production of reducing equivalents to maintain ATP supply. This stimulation is thought to be perturbed when cytosolic Na_i_ is high due to increased Ca_mito_ efflux, potentially compromising ATP_mito_ production and leading to metabolic dysregulation. Increased Na_i_ has been previously shown to affect Ca_mito_; however, whether Na_i_ elevation plays a causative role in energetic mismatching in the hypertrophied and failing heart remains unknown. In this review, we discuss the relationship between elevated Na_i_, NaK ATPase dysregulation and the metabolic phenotype in the contexts of pathological hypertrophy and HF and their link to metabolic flexibility, capacity (reserve) and efficiency that are governed by intracellular ion homeostasis. The development of non-invasive analytical techniques using nuclear magnetic resonance able to probe metabolism *in situ* in the functioning heart will enable a better understanding of the underlying mechanisms of Na_i_ overload in cardiac pathophysiology. They will lead to novel insights that help to explain the metabolic contribution towards these diseases, the incomplete rescue observed with current therapies and a rationale for future energy-targeted therapies.

## Introduction

Cardiovascular disease is the leading cause of mortality worldwide with its incidence projected to rise significantly in the immediate future. There is a clear need for improved understanding of underlying cellular mechanisms which can aid the development of more effective treatments as well as novel techniques for early diagnosis. There is a convincing evidence that myocardial intracellular Na (Na_i_) overload along with metabolic derangement are two important and interconnected pathophysiological features of hypertrophy and heart failure (HF). Na ion homeostasis is regulated by many transporters and membrane pumps [1]. Na/K ATPase (NKA) and its key regulatory protein phospholemman (PLM) play a crucial role in cardiomyocyte transmembrane ion transport and contractility, such that transgenic PLM^3SA^ mice, in which PLM is rendered unphosphorylatable, have chronically elevated Na_i_ and an increased susceptibility to hypertrophy-induced dysfunction [2]. In addition, due to the high ATP demand required for pump activity and its sarcolemmal localization, there is evidence for an association between NKA and metabolism with its ATP supply thought to be supplied preferentially by glycolysis [3–5]. More recent studies have suggested that cytosolic Na regulation also plays an important role in linking mitochondrial Ca-dependent ATP production to mechanical activity and ATP demand due to contractile work [6,8]. Nevertheless, the extent that these metabolic alterations (mismatch in ATP supply–demand) reflect chronic cellular remodelling or arise as a consequence of Na_i_ elevation is not well understood.

## Na pump and Na_i_ regulation in cardiac hypertrophy

In most larger mammalian hearts, with a long action potential, Na_i_ is maintained at ∼4–8 mM [9,10]. In murinae (rats and mice), intracellular Na is significantly elevated (10–20 mM) and this elevated Na is associated with many other adaptations in excitation–contraction (EC) coupling, including a short action potential, a larger recirculating Ca fraction, a dependence on SR Ca release, reduced NCX (sarcolemmal sodium–calcium exchanger) activity, rest potentiation and a negative force–frequency staircase [6].

**Figure 1.**
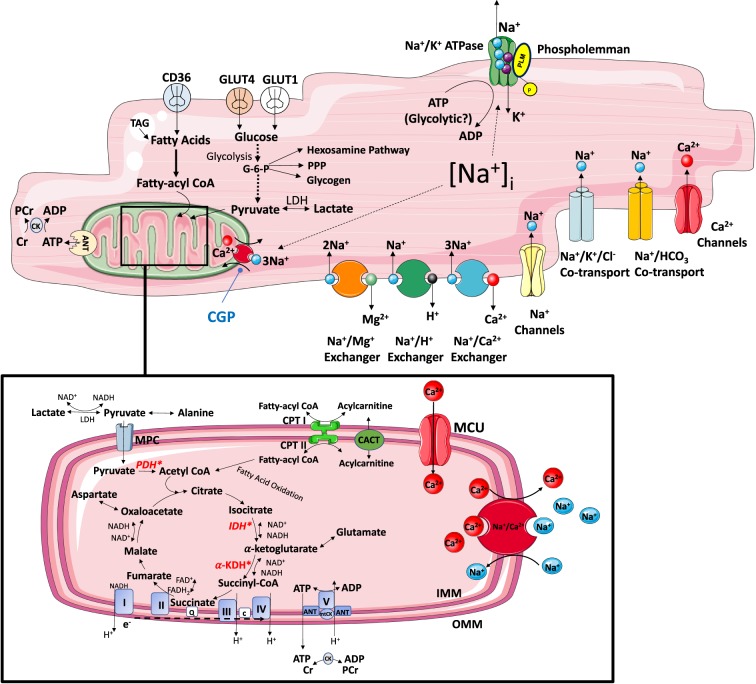
Major Na_i_ influx and efflux pathways and metabolic pathways involved in ATP supply. The delivery of metabolic substrates, their selection and uptake are followed by OXPHOS. It involves electron shuttling from cytosolic to mitochondrial reducing equivalents, transfer of energy by electrons from reducing equivalents to ETC complexes and generation of electrochemical proton (H^+^) gradient within the mitochondrial intermembrane space (respiratory complexes I, II, II, III, IV). The release of H^+^ gradient is coupled to the synthesis of ATP from ADP + P_i_ by F_0_,F_1_-ATPase (complex V), contributing >95% of ATP synthesis under aerobic conditions. The final stage of myocardial ATP supply (phosphotransfer) involves delivery of ATP from mitochondria to sites of use. This involves ADP–ATP exchange across the inner mitochondrial membrane by the adenine nucleotide transporter (ANT) and propagation of local ATP/ADP disequilibria primarily by the creatine kinase (CK). Abbreviations: TAG, triacylglycerol; PCr, phosphocreatine; ANT, adenine nucleotide transporter; GLUT, glucose transporter; CD36, fatty acid transporter; PPP, pentose phosphate pathway; LDH, lactate dehydrogenase; PDH, pyruvate dehydrogenase; CPT, carnitine palmitoyltransferase; CACT, carnitine–acylcarnitine translocase; MCU, mitochondrial calcium uniporter; α-KDH, α-ketoglutarate dehydrogenase; IDH, isocitrate dehydrogenase; mitoCK, mitochondrial creatine kinase; IMM, inner mitochondrial membrane; OMM, outer mitochondrial membrane; Q, quinone pool; c, cytochrome *c*; MPC, mitochondrial pyruvate carrier; e^−^, electrons; CGP, mitochondrial Na–Ca exchanger inhibitor CGP-37157. *Mitochondrial calcium-sensitive dehydrogenases (pyruvate dehydrogenase, isocitrate dehydrogenase and α-ketoglutarate dehydrogenase).

The cell exploits the energy in the transmembrane Na gradient to drive a plethora of Na-dependent membrane transporters moving ions, substrates, amino acids etc. either into (co-transporters/symports) or out of (exchangers/antiports) the cell (Figure 1). The importance of this trans-sarcolemmal inward Na gradient means that its dissipation in various pathologies such as ischaemia/reperfusion [11], hypertrophy or HF [12,13] is highly detrimental. While some of the Na transport processes are electro-neutral, some are electrogenic and hence both respond to, and contribute to, the membrane potential. Most notably, voltage-gated Na channels are crucially important in generating the upstroke of the cardiac action potential. While there are a large number of Na influx pathways, there is only a single quantitatively significant Na efflux pathway responsible for maintaining the transmembrane Na gradient — the Na/K ATPase or Na/K pump (NKA) [14].

**Figure 2.**
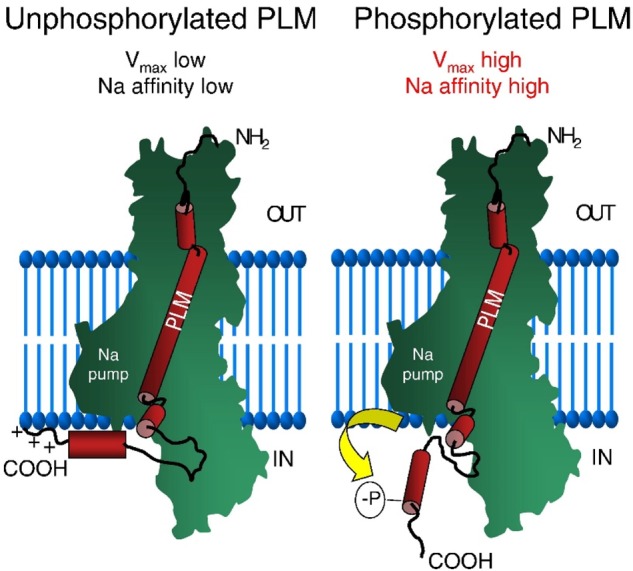
Schematic depiction of a structure–function relationship (regulation) between PLM and Na pump. The cytoplasmic tail of unphosphorylated PLM interacts closely with the membrane and α-subunit of Na pump, whereas phosphorylation alters the association between the pump and PLM by moving the cytosolic arm away from the pump, but not by promoting their dissociation. Phosphorylation or ablation of PLM relieves inhibition of the Na pump by increasing its *V*_max_ and apparent Na affinity. Under stress, phosphorylation of PLM allows the heart to reduce its Na and Ca load and prevents lethal arrhythmias. Adapted from Pavlovic et al. [15].

The activity of the NKA is regulated by FXYD1, or PLM, the principal sarcolemmal target of protein kinases A and C (Figure 2) [15]. As such, PLM is required for the dynamic control of Na_i_ during increases in heart rate or during disease and plays a vital role in Na regulation during ‘fight or flight’ [14]. Under physiological conditions, NKA is the only quantitatively significant efflux pathway of Na out of the myocyte (NCX and Na/HCO_3_/Cl symporter, in principle, can reverse and efflux Na) [16] (Figure 1).

A hallmark of cardiac hypertrophy and failure is an elevation of Na_i_. There is an abundant literature on this phenomenon, although absolute values of measured Na_i_ are often dissimilar, probably owing to methodological differences as summarized in Table 1. Elevation in Na_i_ may contribute to the negative force–frequency relationship, slowed relaxation and arrhythmias [17]. While a component of the elevation of Na_i_ may reflect an increase in Na influx [10], there is a large body of evidence showing that Na/K pump function may also be compromised [2,12,13,18]. Specifically, in cardiac hypertrophy, many studies have shown that NKA pump function, and/or expression, is reduced [2,12,13,18,19]. Cardiac Na_i_ can also be elevated by several other factors such as hypothermia [16] or by increased intracellular pH via enhanced NHE (sodium–proton exchanger) activity, for example, following ischaemia–reperfusion injury [20].

**Table 1:** Summary of studies quantifying bulk cytosolic [Na]i in the myocyte under both physiological and pathophysiological conditions across various mammalian species

Species	[Na]_i_ (mM)	Method used	Reference
Human	8.0	SBFI (sodium-binding benzofuran isophthalate)-loaded muscle strips paced at 0.25 Hz	[17]
Human LVH	14.2	Na-selective microelectrodes; muscle strips at rest	[67]
Human failing	12.1	SBFI-loaded muscle strips paced at 0.25 Hz	[17]
Human MVD	11.8	Na-selective microelectrodes; muscle strips at rest	[67]
Sheep	5–6.4	Na-selective microelectrodes; Purkinje fibres at 1 Hz and at rest	[68,69]
	5.8–7.9	Na-selective microelectrodes; muscle strips at rest	[70]
Dog	8.9–10.4	Na-selective microelectrodes; Purkinje fibres at rest and 1 Hz	[71]
Guinea pig	4.7–8.0	Na-selective microelectrodes; muscle strips at rest	[67,70,72,73,74]
	6.4	^23^Na NMR; isolated perfused heart	[75]
	5.1–5.2	SBFI-loaded myocytes at rest	[9,76]
Guinea pig LVH	12.1	Na-selective microelectrodes; muscle strips at rest	[67]
	12.8	^23^Na NMR; isolated perfused heart	[75]
Guinea pig failing	16.8	SBFI-loaded myocytes at rest	[77]
Ferret	7.8	Na-selective microelectrodes; muscle strips at rest	[78]
Ferret RVH	8.0	Na-selective microelectrodes; muscle strips at rest	[78]
Rabbit	7.2	Na-selective microelectrodes; muscle strips at 0.5 Hz	[6,10]
	3.8–4.5	SBFI-loaded myocytes at rest	[10,79]
Rat	12.7	Na-selective microelectrodes; muscle strips at 0.5 Hz	[6]
	8.5–30	Na-selective microelectrodes; myocytes at rest	[80]
	5.1–21	SBFI-loaded myocytes at rest	[9,10,79,81]
	17.5	^23^Na NMR; isolated perfused arrested hearts	[82]
Mouse	11.6	^23^Na NMR; isolated perfused heart	[51]
Mouse	14	SBFI-loaded myocytes at rest	[2]
Mouse LVH	23	SBFI-loaded myocytes at rest	[2]

## ATP supply–demand matching in the heart

Fine control of ATP-generating pathways in mitochondria and cytosol are critical to meet the energy demands of cardiac muscle. Supply must be matched to demand as failure to provide an adequate amount of ATP causes a decrease in cellular free energy leading to mechanical failure. The heart utilizes more energy than any other organ — with 2% of its total ATP reserves consumed per beat, it turns over its total ATP pool in less than 1 min and utilizes 6 kg of ATP every day [21–23]. This enormous energy demand is related primarily to ATP-dependent processes driving EC coupling [24]. About 70–75% of total intracellular ATP is used for force generation powering work output, with the remaining 25–30% is used for basal metabolism [25–27]. In terms of force generation, it is estimated that the actomyosin ATPase accounts for 76%, SERCA (sarcoendoplasmic reticulum Ca^2+^ ATPase) 15% and NKA for 9% of ATP utilization [27].

To synthesize the ATP required for normal function, the adult heart converts chemical energy primarily stored in free fatty acids (FFAs) (60–90%) and pyruvate (derived from glucose and lactate 10–40%) into mechanical energy for contraction [28]. The delivery of metabolic substrates, their selection, uptake and oxidation to generate acetyl-CoA for tricarboxylic acid (TCA) cycle entry and ATP generation in the electron transport chain (ETC) comprises three stages of myocardial ATP supply as summarized in Figure 1. However, cardiac workload varies constantly, including several-fold increase in cardiac output during exercise, thus requiring rapid and continuous matching of ATP supply to demand. This renders the heart a metabolic omnivore, giving it a high degree of substrate flexibility to rapidly switch substrate preference and utilization [28]. The apparent opposing relationship between carbohydrates and FFAs in the heart is, in part, due to the Randle (glucose–fatty acid) cycle, thus optimizing energy supply by avoiding energetic inefficiency and ‘waste’ [29].

## The failing heart

First identified in the early 20th century, and now a well-established energy starvation hypothesis, it is proposed that maladaptive metabolic remodelling precedes, initiates and maintains adverse contractile dysfunction in hypertrophy and HF [23,24]. Advances in analytical technologies and understanding of metabolic mechanisms have improved our insights into the phenomenon and helped to classify metabolic alterations leading to myocardial energy starvation into those related to substrate utilization, intermediary metabolism and energetics. Using *in vivo* ^31^P nuclear magnetic resonance (NMR), Neubauer [23] found that the myocardial phosphocreatine-to-ATP ratio (PCr:ATP) can be used as a reliable prognostic indicator of dilated cardiomyopathy (DCM) where 44% of DCM patients with a PCr:ATP of <1.6 died of cardiovascular causes vs. 5% with a PCr:ATP of >1.6. Cardiac hypertrophy induces a switch in substrate utilization from dominant FFA oxidation towards carbohydrate utilization which is similar to the foetal metabolic phenotype [24,30–32]. The onset of this switch (and thereby the stage at which it could potentially be targeted therapeutically) is currently debated as numerous studies suggest that ATP levels are sustained during the early stages of remodelling and only decrease (30–40%) during advanced stages of HF [33–37]. There have also been numerous preclinical studies as well as clinical data inferring mitochondrial respiratory impairment (complex activities and/or altered expression of the ETC complexes, ATP synthase and adenine nucleotide translocase) in hypertrophy and HF [38–40].

EC coupling, specifically Ca handling by SERCA, has also been linked to the time course of metabolic alterations during hypertrophy: SERCA preferentially uses glycolytically derived ATP over OXPHOS (oxidative phosphorylation) [41] and therefore switching to a more glycolytic phenotype during hypertrophy, and HF could reflect increased SERCA activity to sustain adequate Ca homeostasis. NKA pump also requires glycolysis for normal Na homeostasis, potentially due to preferential fuelling of NKA by cytosolic glycolytically derived ATP and its spatial proximity to the pump [4,42,43]. However, the substrate switch and energetic deficit alone cannot explain Na accumulation observed in hypertrophy and failure. The debate is similar to the arguments about Na elevation in ischaemia and revolves around energetic inhibition of the NKA: the substrate switch from fatty acids to glucose leads to impaired energetic reserve and decline in cytosolic ATP, thus limiting the energy supply to the pump leading to Na accumulation. However, it has been previously shown that even during severe metabolic stress such as ischaemia, intracellular Na rises at a time when the total ATP concentration greatly exceeds the *K*_m_ for the pump (∼0.1–0.8 mmol/l) and the free energy of ATP exceeds that required for pump activity (∼44 kJ/mol) [44].

## Myocardial Na_i_ elevation and metabolic remodelling: the chicken or the egg?

In spite of significant evidence to support the concomitance of Na_i_ overload and metabolic remodelling during cardiac hypertrophy and HF, there have been very few studies investigating the interaction between these pathophysiological events. Using isolated rat mitochondria, Iwai et al. [45] demonstrated that increasing extramitochondrial Na (Na_ex_) from physiological (12.5 mM) to supraphysiological (≥25 mM) concentrations significantly reduced state 3 respiration, suggesting reduced mitochondrial ATP supply as well as reduced mitochondrial membrane potential. However, the present study offered no insights into the mechanism underlying the effect of Na_i_ overload on whole cell metabolism.

A series of studies focusing on the mitochondrial transport of Na and Ca and its relationship with mitochondrial ATP production showed a stimulation of mitochondrial ATP production by Ca; however, the mitochondrial Ca transport kinetics and its regulation by Na_i_ are still not completely understood [45–48]. The majority of Ca_mito_ uptake is by the Ca_mito_ uniporter (MCU), while the Na/Ca_mito_ exchanger (NCLX) is thought to be the predominant mechanism for Ca extrusion [49] (Figure 1). The impact of Na_i_ on Ca_m_ has been elucidated by Cox and Matlib [46] using fura-2 to measure Ca_m_ in isolated cardiac mitochondria from healthy rabbits. Mitochondria incubated with increasing concentrations of Na_ex_ using NaCl in the physiological range showed reduced Ca_mito_ as well as reduced NADH production and state 3 respiration. On the other hand, inhibition of NCLX with three inhibitors (from highest to lowest potency: CGP-37157 > clonazepam > d-*cis*-diltizam) and the MCU inhibitor ruthenium red substantially increased Ca_m_, NADH production and state 3 respiration in a dose-dependent manner. This study supported the findings of Iwai et al. [45] and the hypothesis that Na_i_ overload dysregulates ATP supply–demand matching.

However, this study did not provide information on the beat-to-beat kinetics of Ca_m_ transport and its relation to mitochondrial energy production.

Isolated mitochondria experiments should be treated with caution, given the measurements are performed in the absence of important ATP sinks (myosin ATPase, NKA and SERCA) and substrate utilization pathways (glycolysis and β-oxidation). More recently, Maack et al. [50] used isolated guinea pig cardiomyocytes to measure Ca_i_ and Ca_m_ during systole and diastole as well as NADH, thereby providing insights into the beat-to-beat regulation of Ca_m_ during increased Na_i_. This study showed that both systolic and diastolic Ca_m_ are significantly reduced by Na_i_ elevation. Correspondingly, the percentage of NAD(H) in the reduced form was maintained at ∼62% in the control group, but was significantly lower in the high Na_i_ group. In spite of these changes in Ca and [NADH], Na_i_ elevation did not affect the mitochondrial membrane potential (ΔΨ_m_). Furthermore, NCLX inhibition by CGP-37157 was shown to significantly elevate diastolic Ca_m_. As these effects were not altered by ruthenium red inhibition of the MCU, it is likely a consequence of increased Ca_m_ extrusion via NCLX on a beat-to-beat basis. The outcome of this study further supported the argument that Na_i_ is an important regulator of cardiac bioenergetics. However, it remains unclear whether this is truly reflective of a regulatory mechanism in the beating heart and, if so, which metabolic pathways are most affected by Na_i_ overload.

## Measuring Na_i_ overload in the beating heart

The studies examining the impact of Na_i_ on mitochondrial ATP provision published to date are subject to major experimental caveats, thus making direct mechanistic translation to *in situ* perfused and *in vivo* myocardium difficult. Specifically, these studies lack integrated experimental approaches as they have been limited to isolated organelles and cells at subphysiological temperatures with limited metabolic readouts.

To elucidate the importance of the link between Na_i_ and ATP supply–demand matching in the beating heart, experimental models are required either *ex vivo* or *in vivo* where the heart is perfused under physiologically relevant conditions and where Na_i_ elevation can be induced and reliably measured. To elucidate concomitant changes in substrate metabolism or energetics, it is also necessary to be able to quantify a wide range of metabolites involved in energy homeostasis in these models. We have previously applied and validated techniques able to measure intracellular Na_i_ in the Langendorff perfused mouse [51,52] or rat heart preparations [53] using NMR.

^23^Na NMR has historically been used to distinguish the small intra Na_i_ versus large extracellular Na_e_ pools employing paramagnetic shift reagents such as Tm(DOTP) [thulium (III) 1,4,7,10-tetraazacyclododecane-1,4,7,10-tetra(methylenephosphonate)] [51] to separate the two. However, these reagents are efficient chelators of Ca and Mg leading to modified ion homeostasis and reduced cardiac contractility [51]. As a result, shift reagents exhibit significant toxicity precluding their use *in vivo* and question their validity for measuring Na_i_ *ex vivo*. In contrast, multiple quantum-filtered ^23^Na NMR, which exploits the quadrupolar property of the ^23^Na nucleus, has shown potential to probe intra and extracellular pools of Na in the *absence* of shift reagent and therefore under more physiological conditions [54,55]. We have previously investigated the use of these techniques in the perfused mouse heart where we were able to measure elevated intracellular Na in response to the cardiac glycoside ouabain as well as in response to modified buffer compositions, for example, in the absence of K, Ca or Mg. We were further able to verify previous studies showing that the PLM^3SA^ mouse has a chronic elevation of basal Na_i_ compared with wild-type hearts. Crucially, NMR is also able to probe cardiac energetics by ^31^P NMR in the same hearts where it is possible to measure the concentrations of ATP, PCr, P_i_, intracellular pH as well as PCr:ATP ratio and thereby derive estimates of the Gibb's free energy. Newly emerging techniques such as metabolomics also enable end-point measurements of metabolites in snap frozen extracted myocardial tissue and coronary effluent using either high-resolution NMR or mass spectrometry [53,56,57].

**Figure 3.**
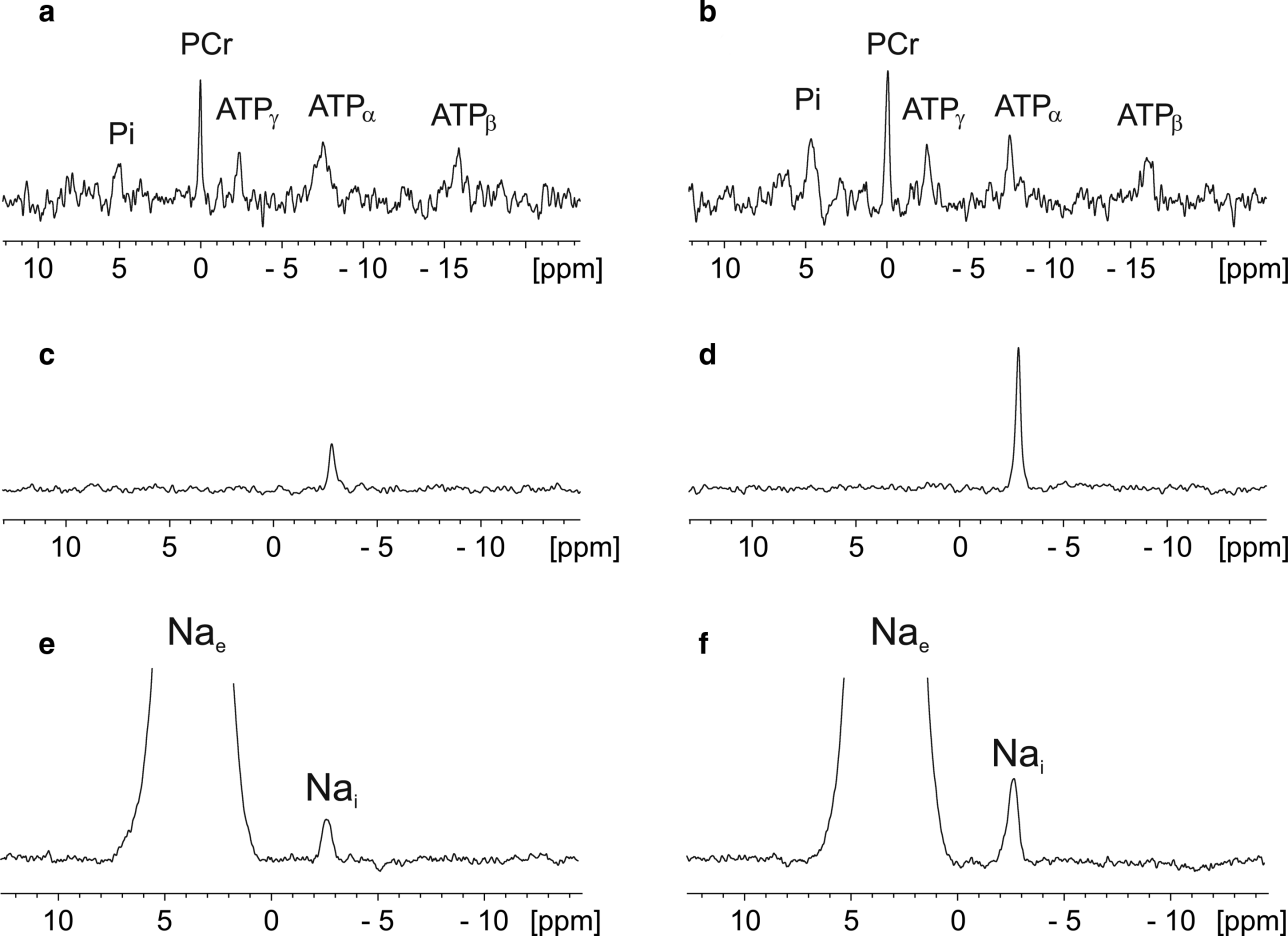
Representative ^31^P NMR spectra, triple-quantum-filtered ^23^Na and conventional 1D ^23^Na NMR spectra from perfused control and hypertrophied mouse hearts. The spectra displayed in the left panel (**a**, **c** and **e**) are from a control heart, while those displayed in the right panel (**b**, **d** and **f**) are from a hypertrophied heart. All NMR data were acquired as previously described [51] using a Bruker Avance III 400 MHz wide-bore spectrometer. Briefly, **a** and **b** show ^31^P spectra, **c** and **d** show triple-quantum-filtered (TQF) ^23^Na NMR spectra, while **e** and **f** show conventional single-quantum ^23^Na NMR spectra acquired at the end of the perfusion during infusion of 5 mM Tm(DOTP).

Figure 3 shows the example NMR spectra of two different Langendorff perfused mouse hearts acquired using our previously reported NMR protocols. The left-hand panel displays data from a wild-type control mouse heart with normal baseline Na_i_, while the right-hand panel displays data from a hypertrophic mouse heart subject to aortic constriction [2]. The dry weight of the control heart was 30 mg while that of the banded heart was 58 mg measured at the end of the experiment. Figure 3a,b shows the ^31^P NMR spectra acquired with baseline function. Figure 3c,d shows the triple-quantum-filtered ^23^Na NMR spectra acquired with baseline function. Figure 3e,f shows the conventional single-quantum-filtered ^23^Na NMR spectra acquired at the end of the experiment following infusion of 5 mM Tm(DOTP) to shift the large extracellular Na_e_ signal and enable quantification of the small intracellular Na_i_ signal. Our previous work suggested that the TQF signal in Figure 3c,d consists of a contribution both from the intracellular and extracellular pools of Na, but that the large bulk isotropic signal from the buffer is largely suppressed. These experiments highlight the ability of such NMR techniques to probe both cardiac energetics using ^31^P NMR and Na_i_ using triple-quantum-filtered ^23^Na NMR in the same preparation [51]. The data presented here also highlight experimental challenges in quantifying Na_i_ in these hearts. Total myocardial Na_i_ is clearly elevated under conditions of hypertrophy; however, so too is the tissue mass and intracellular volume [51]. Absolute quantification of such data is subject to many experimental assumptions including a phenomenological scaling factor for the NMR observability of Na_i_ and a scaling factor to estimate intracellular volume. Despite obvious limitations in the methodology, NMR offers unique insights into Na ion homeostasis and cardiac energetics under both physiological and pathophysiological conditions. Additionally, there has resurgence in interest applying MRI techniques for imaging Na distribution *in vivo* [58]. ^23^Na is the second most sensitive nucleus for *in vivo* detection by NMR after ^1^H; however, sensitivity and spatial resolution remain an issue as well as the ability to separate intra- versus extracellular pools of Na which is also challenging.

## Therapeutic potential

Na_i_ has inadvertently been a known therapeutic target in HF for the last 200 years, and the established example of *in vivo* use of Na_i_ modulation is the administration of cardiac glycosides (such as digoxin) which are potent inhibitors of NKA. Cardiac glycosides elevate Na_i_ and lead to a positive inotropic response (due to release of Ca) that can vary considerably between species. They are steroidal-like compounds found endogenously under normal conditions (e.g. ouabain, digoxin and bufalin) and are elevated in patients with renal failure [59] and HF [60]. However, their clinical use for the treatment of HF is a cautionary tale and limited due to their energetically costly as well as pro-arrhythmic properties [61]. Nevertheless, they remain useful tool for elevating Na_i_ in experimental models [51–53,56,62]. In spite of the substantial *in vitro* and preclinical evidence to support the targeting of the substrate switch therapeutically, there has been limited successes that have been translated into the clinic. For example, sodium dichloroacetate (pyruvate dehydrogenase kinase inhibitor) appeared to improve contractile performance in 10 HF patients, but a vehicle control group was not included in this study [63,64]. Trimetazadine is currently prescribed for longer term inhibition of FFA oxidation and has been shown to reduce angina and improve cardiac function in patients with DCM [65,66], although these improvements were modest. Given the limited clinical success of targeting substrate utilization to date, it is important to continue to evaluate the potential of targeting other aspects of cardiac metabolism, such as intermediary pathways leading to ATP supply. This could also help identify the role metabolic remodelling plays in transition from pathological hypertrophy towards HF. The question remains whether early prevention of myocardial Na_i_ elevation could either prevent the origin or alter the course of metabolic derangement in pathological hypertrophy leading to energy starvation and cardiac death. This hypothesis warrants further study including the ongoing development of therapeutics that target these interconnected pathophysiological events.

## Abbreviations

Ca_mito_: mitochondrial calcium
DCM: dilated cardiomyopathy
EC: excitation–contraction
ETC: electron transport chain
FFA: free fatty acids
HF: heart failure
MCU: mitochondrial calcium uniporter
Na_ex_: extramitochondrial sodium
Na_i_: intracellular sodium
NCLX: mitochondrial sodium–calcium exchanger
NCX: sarcolemmal sodium–calcium exchanger
NKA: sodium/potassium ATPase
NMR: nuclear magnetic resonance
OXPHOS: oxidative phosphorylation
PCr:ATP: phosphocreatine-to-ATP ratio
PLM: phospholemman
SBFI: sodium-binding benzofuran isophthalate
SERCA: sarcoendoplasmic reticulum Ca^2+^ ATPase
Tm(DOTP): thulium (III) 1,4,7,10-Tetraazacyclododecane-1,4,7,10-tetra(methylenephosphonate)
